# Blockage of Glyoxalase I Inhibits Colorectal Tumorigenesis and Tumor Growth via Upregulation of STAT1, p53, and Bax and Downregulation of c-Myc and Bcl-2

**DOI:** 10.3390/ijms18030570

**Published:** 2017-03-09

**Authors:** Yuan Chen, Lei Fang, Jiali Zhang, Gefei Li, Mengni Ma, Changxi Li, Jianxin Lyu, Qing H. Meng

**Affiliations:** 1 Key Laboratory of Laboratory Medicine, Ministry of Education of China, Zhejiang Provincial Key Laboratory of Medical Genetics, School of Laboratory Medicine and Life Sciences, Wenzhou Medical University, Wenzhou 325035, China; 15057766065@163.com (Y.C.); 15158652395@163.com (L.F.); 15057765887@163.com (J.Z.); ligefei1992@163.com (G.L.); mamengni123@163.com (M.M.); 15967411311@163.com (C.L.); 2 Department of Laboratory Medicine, The University of Texas MD Anderson Cancer Center, Houston, TX 77030, USA

**Keywords:** glyoxalaseI, colorectal cancer, knockdown, signal transducer and activator of transcription 1, p53

## Abstract

GlyoxalaseI (GLOI) is an enzyme that catalyzes methylglyoxal metabolism. Overexpression of GLOI has been documented in numerous tumor tissues, including colorectal cancer (CRC). The antitumor effects of GLOI depletion have been demonstrated in some types of cancer, but its role in CRC and the mechanisms underlying this activity remain largely unknown. Our purpose was to investigate the antitumor effects of depleted GLOI on CRC in vitro and in vivo. RNA interference was used to deplete GLOI activity in four CRC cell lines. The cells’ proliferation, apoptosis, migration, and invasion were assessed by using the Cell Counting Kit-8, plate colony formation assay, flow cytometry, and transwell assays. Protein and mRNA levels were analyzed by western blot and quantitative real-time PCR (qRT-PCR), respectively. The antitumor effect of GLOI depletion in vivo was investigated in a SW620 xenograft tumor model in BALB/c nude mice. Our results show that GLOI is over-expressed in the CRC cell lines. GLOI depletion inhibited the proliferation, colony formation, migration, and invasion and induced apoptosis of all CRC cells compared with the controls. The levels of signal transducer and activator of transcription 1 (STAT1), p53, and Bcl-2 assaciated X protein (Bax) were upregulated by GLOI depletion, while cellular homologue of avian myelocytomatosis virus oncogene (c-Myc) and B cell lymphoma/lewkmia-2 (Bcl-2) were downregulated. Moreover, the growth of SW620-induced CRC tumors in BALB/c nude mice was significantly attenuated by GLOI depletion. The expression levels of STAT1, p53, and Bax were increased and those of c-Myc and Bcl-2 were decreased in the GLOI-depleted tumors. Our findings demonstrate that GLOI depletion has an antitumor effect through the STAT1 or p53 signaling pathways in CRC, suggesting that GLOI is a potential therapeutic target.

## 1. Introduction

Colorectal cancer (CRC) is the third most common cancer in the U.S. and the third leading cause of cancer mortality among both men and women [1]. The current standard of CRC management is surgery with adjunctive chemotherapy and/or radiotherapy, but targeted therapy and immune therapy are being introduced into the clinical setting [2,3]. Although these emerging comprehensive approaches have improved therapeutic efficacy, the rates of recurrence and metastasis remain high, resulting in an overall low survival rate and poor prognosis [2,3,4,5,6]. It is thus critical to find new therapeutic approaches that improve patient outcomes.

The glyoxalase system is a set of enzymes that carry out the detoxification of methylglyoxal mainly produced during glycolysis. It is consists of two key enzymes: glyoxalase I (GLOI) which catalyzes the formation of *S*-d-lactoylglutathione from methylglyoxal, and glyoxalase II which catalyzes the hydrolysis of *S*-d-lactoylglutathioneto d-lactate. Tumor cells have high glycolytic activity. The increased activity of the detoxification system in cancerous cells makes this pathway a viable target for developing novel anticancer agents [7,8,9,10,11].

$$\mathrm{Methylglyoxal}+\mathrm{GSH}\overset{\mathrm{glyoxalase}\;I}{\to }S-\text{D}-\mathrm{lactoylglutathione}\overset{\mathrm{glyoxalase}\;\mathrm{II}}{\to }\text{D}-\mathrm{lactate}+\mathrm{GSH}$$

Overexpression of GLOI has been documented in numerous tumor tissues and cells, including colon, breast, prostate, lung, stomach, endometrial, and ovarian cancers [11,12,13,14,15,16,17,18,19]. GLOI overexpression has been associated with cancer cell survival and resistance to chemotherapeutic agents [20,21]. For example, GLOI was overexpressed in human leukemia cells resistant to antitumor agents [15]. GLOI overexpression enhanced resistance to anti-tumor agents such as etoposide and doxorubicin [20]. Silencing of GLOI in tumors with high rates of glycolysis and methylglyoxal formation led to accumulation of methylglyoxal and cytotoxicity [22,23]. These findings suggest that GLOI plays a crucial role in tumorigenesis.

Although GLOI has been shown to have biological roles in some types of cancer, its role in colon cancer remains largely unknown. In this study, we investigated the effects of GLOI on CRC cells in vitro and in vivo and the molecular mechanisms underlying these effects.

## 2. Results

### 2.1. Expression of GLOI in Human CRC Cells

**Figure 1:**
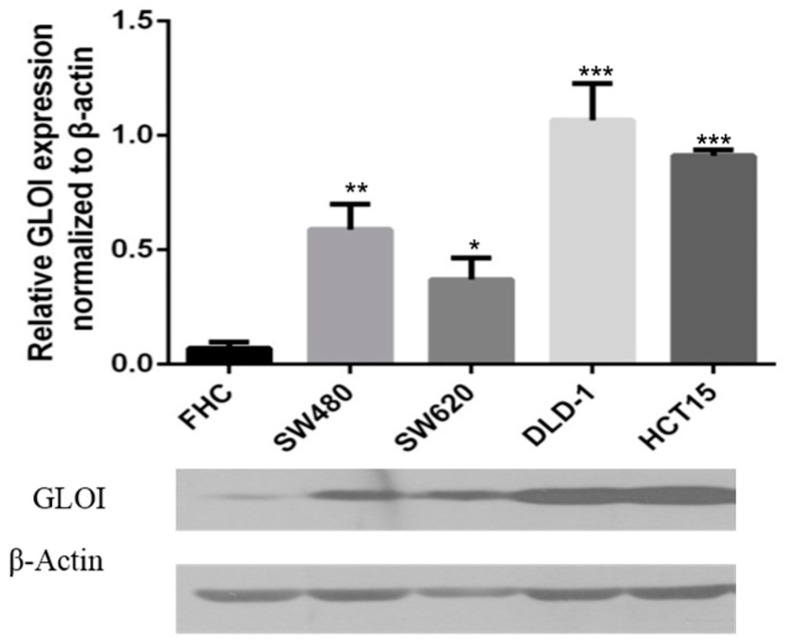
Glyoxalase I (GLOI) is over-expressed in colorectal cancer (CRC) cell lines. GLOI expression in normal colon cells (FHC) and CRC cell lines (SW480, SW620, DLD-1, and HCT-15) was determined by western blot. β-Actin was used as the internal control. * *p* < 0.05, ** *p* < 0.01, *** *p* < 0.001. All data are representative of three independent experiments (*n* = 3).

GLOI expression was significantly higher in CRC cells SW480 (*p* < 0.01), SW620 (*p* < 0.05), DLD-1 (*p* < 0.001), and HCT-15 (*p* < 0.001) than in normal FHC colon cells (Figure 1). Levels of GLOI protein in the CRC cells ranged from 50% to 100% higher than those in FHC cells.

### 2.2. GLOI Knockdown Suppressed GLOI mRNA, Protein, and Enzyme Activity in Human CRC Cell Lines

**Figure 2:**
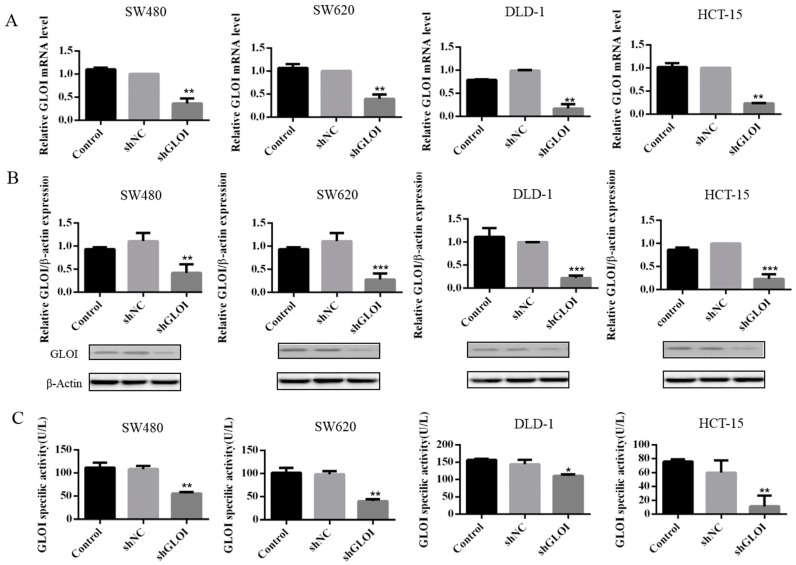
GLOI knockdown suppresses expression of GLOI mRNA, protein, and enzyme activity in colorectal cancer (CRC) cells. The expression of GLOI mRNA (**A**), GLOI protein (**B**), and GLOI enzyme activity (**C**) was lower in SW480, SW620, DLD-1, and HCT-15 CRC cells transfected with short hairpin GLOI (shGLOI) than in cells transfected with short hairpin empty vector (shNC). * *p* < 0.05, ** *p* < 0.01, *** *p* < 0.001. All data are representative of three independent experiments (*n* = 3).

Both GLOI mRNA (Figure 2A) and protein (Figure 2B) levels were significantly lower in SW480, SW620, DLD-1, and HCT-15 CRC cells transfected with shGLOI than in those transfected with shNC (*p* < 0.01 to 0.001). Similarly, GLOI enzyme activity was markedly lower in CRC cells transfected with shGLOI than in those transfected with shNC (*p* < 0.05 to 0.01) (Figure 2C).

### 2.3. Inhibition of GLOI Expression in Human CRC Cell Lines Decreased Proliferation, Colony Formation, Migration, and Invasion, and Induced Apoptosis

**Figure 3:**
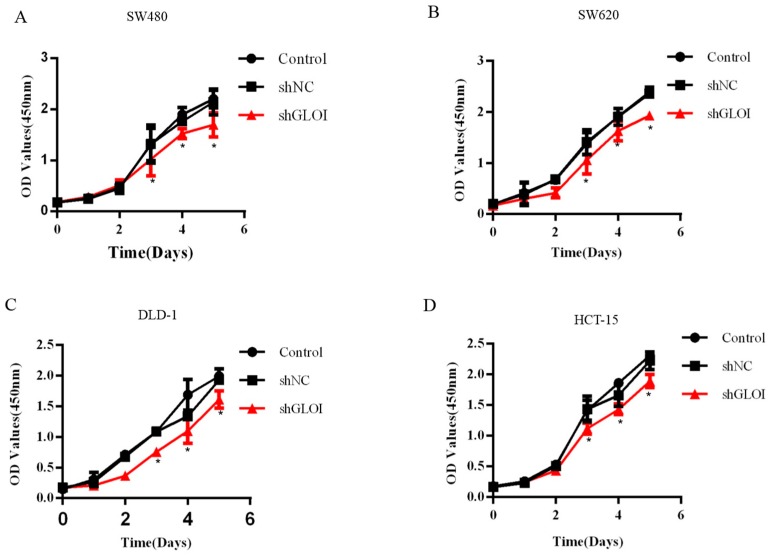
GLOI knockdown inhibits colorectal cancer (CRC) cell viability. (**A**–**D**) SW480, SW620, DLD-1, and HCT-15 CRC cells were transfected with shGLOI or shNC. The growth of the cells was monitored for 5 days. Optical density (OD) * *p* < 0.05. All data are representative of three independent experiments (*n* = 3).

**Figure 4:**
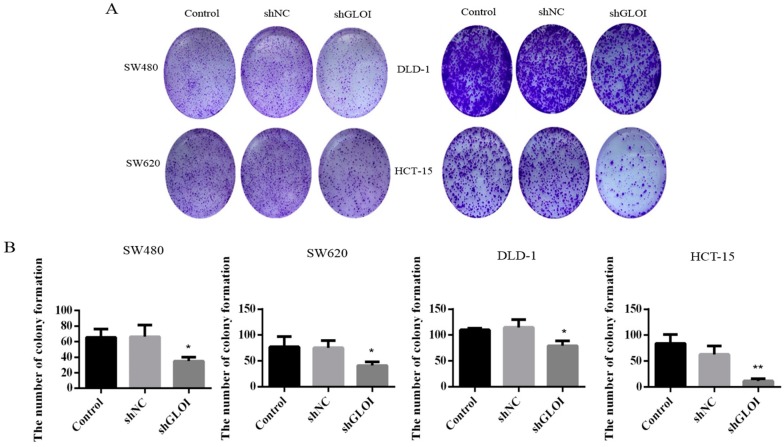
GLOI knockdown suppresses colony formation by colorectal cancer (CRC) cells. (**A**) SW480, SW620, DLD-1, and HCT-15 CRC cells were transfected with shGLOI or shNC (empty vector), and colony formation was monitored. (**B**) Numbers of colonies formed by CRC cells were quantified for comparison. * *p* < 0.05, ** *p* < 0.01. All data are representative of three independent experiments (*n* = 3).

**Figure 5:**
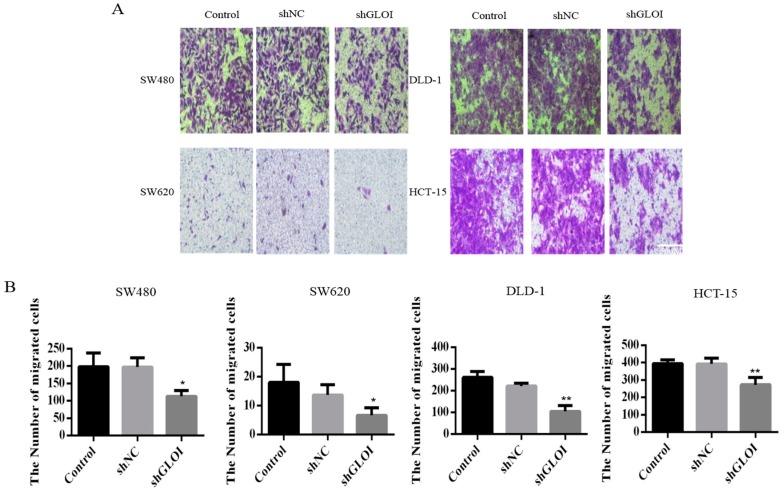
GLOI knockdown inhibits migration of colorectal cancer (CRC) cells. (**A**,**B**) SW480, SW620, DLD-1, and HCT-15 CRC cells were transfected with shGLOI or shNC (empty vector) and their migration was evaluated by their penetration of a transwell insert membrane. * *p* < 0.05, ** *p* < 0.01. All data are representative of three independent experiments (*n* = 3).

**Figure 6:**
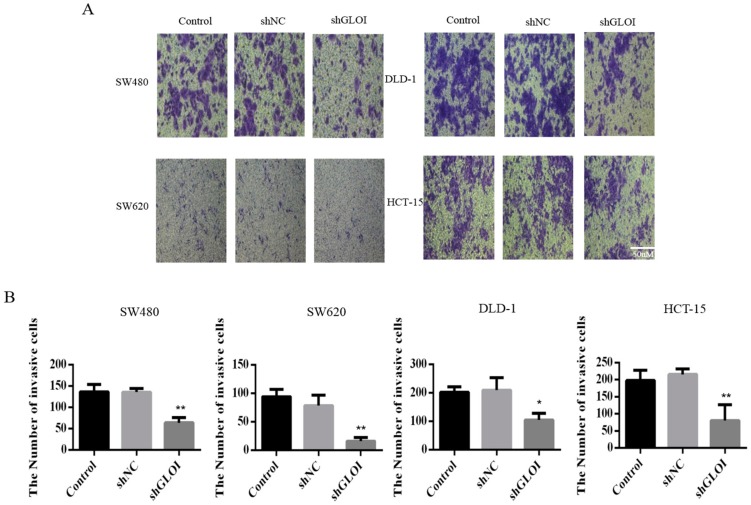
GLOI knockdown inhibits invasion by colorectal cancer (CRC) cells. (**A**,**B**) SW480, SW620, DLD-1, and HCT-15 CRC cells were transfected with shGLOI or shNC (empty vector) and their invasiveness evaluated by their penetration of a transwell insert membrane coated with matrigel. * *p* < 0.05, ** *p* < 0.01. All data are representative of three independent experiments (*n* = 3).

GLOI downregulation significantly inhibited the growth of human CRC cells at day 3, day 4, and day 5 (*p* < 0.05) (Figure 3). GLOI depletion reduced the numbers of colonies formed by human CRC cells to about 60% of the numbers formed by shNC cells (*p* < 0.05 to 0.01) (Figure 4A,B). Moreover, the numbers of GLOI-depleted CRC cells that migrated through a transwell insert membrane were markedly lower than those of shNC-transfected cells (*p* < 0.05 to 0.01) (Figure 5A,B). Similarly, the numbers of GLOI-depleted CRC cells penetrating the membrane in the matrigel transwell assay were also dramatically lower than the numbers of shNC-transfected cells (*p* < 0.05 to 0.01) (Figure 6A,B).

**Figure 7:**
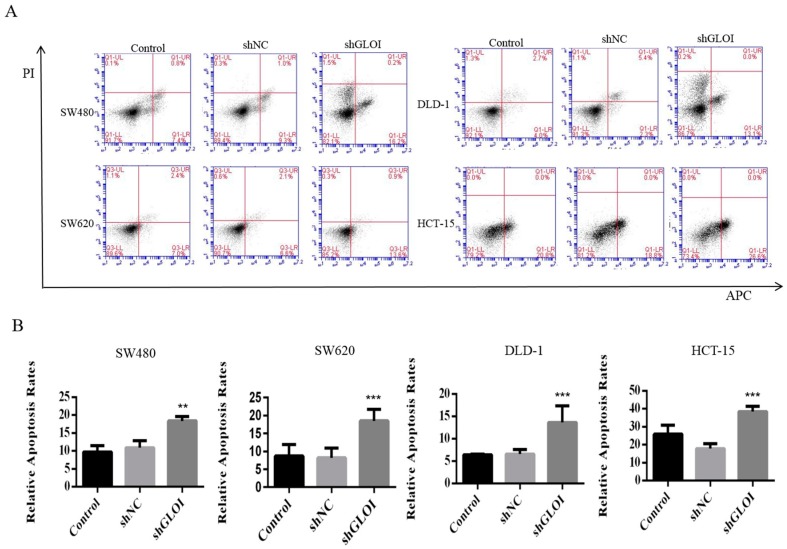
GLOI knockdown increases apoptosis in colorectal cancer (CRC) cells. (**A**) SW480, SW620, DLD-1, and HCT-15 CRC cells were transfected with shGLOI or shNC. The apoptotic cells were detected by annexin V–propidium iodide (PI) flow cytometry. (**B**) The apoptotic rate of each group of cells was quantified. ** *p* < 0.01, *** *p* < 0.001. All data are representative of three independent experiments (*n* = 3).

Cell apoptosis rate was increased by GLOI depletion in CRC cells. The apoptosis rates were 6.1% higher in shGLOI-transfected SW480cells than in shNC-transfected cells (*p* < 0.01), 6.1% higher in SW620 (*p* < 0.001), 5.4% higher in DLD-1 (*p* < 0.001), and 7.8% higher in HCT-15 (*p* < 0.001) (Figure 7A,B).

### 2.4. Inhibition of GLOIexpression in the Human CRC Cell Lines Upregulated STAT1, p53, and Bax Protein Expression While Downregulating c-Myc and Bcl-2expression

**Figure 8:**
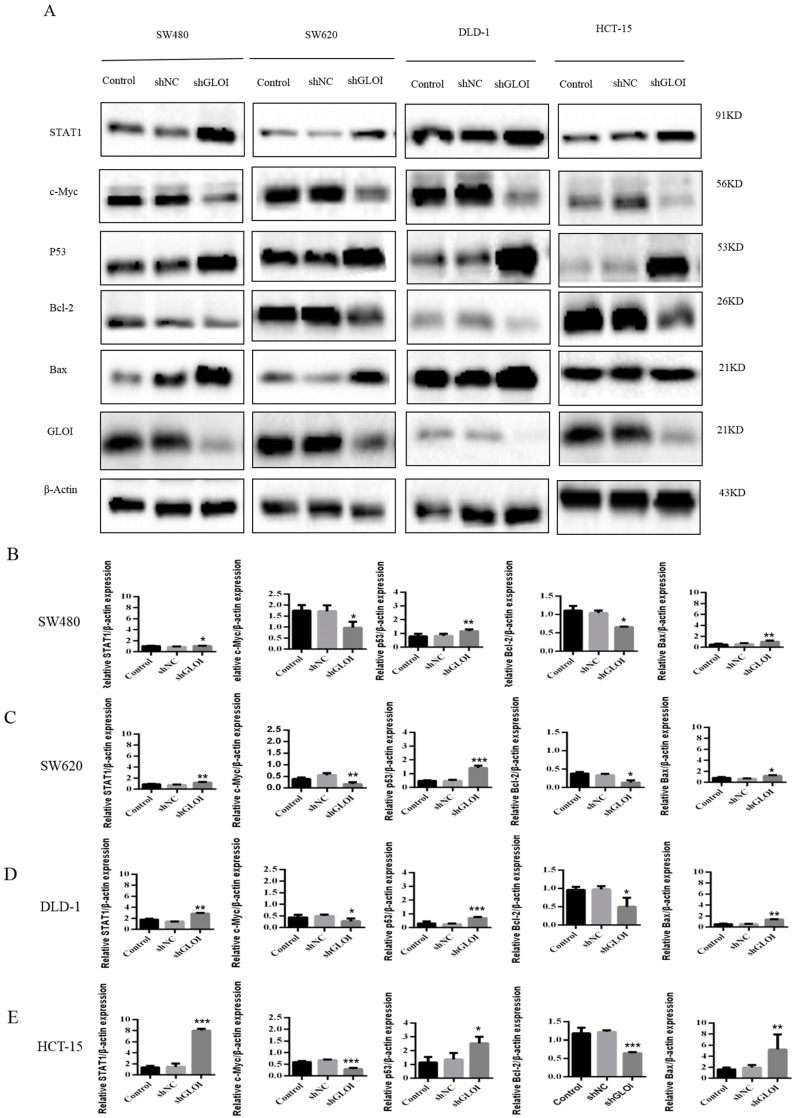
GLOI knockdown alters expression levels of STAT1 or p53 signal pathway proteins in colorectal cancer (CRC) cells. SW480, SW620, DLD-1, and HCT-15 CRC cells were transfected with shGLOI or shNC. The expression levels of signal transducer and activator of transcription 1 (STAT1), p53, c-Myc, Bcl-2, and Bax were determined in the CRC cells by (**A**) western blot and (**B**–**E**) quantification of results. * *p* < 0.05, ** *p* < 0.01, *** *p* < 0.001. All data are representative of three independent experiments (*n* = 3).

STAT1expression was approximately 2-fold higher in shGLOI-transfected HCT-15 cells (*p* < 0.001), 1.5-foldhigher in DLD-1 (*p* < 0.01), 1.5-fold higher in SW620 (*p* < 0.01), and 1.2-fold higher in SW480 (*p* < 0.05) than in the corresponding shNC-transfected cells (Figure 8A–E). p53 expression was 3-fold higher in shGLOI-transfected SW620 cells (*p* < 0.001), 2-fold higher in DLD-1 (*p* < 0.001), 2-fold higher in HCT-15(*p* < 0.05), and 1.5-fold higher inSW480 (*p* < 0.01) than in the shNC-transfected cells (Figure 8A–E). Similarly, Bax, the downstream target of p53, was also up-regulated in shGLOI-transfected CRC cells (*p* < 0.05 to 0.01) (Figure 8A–E). In contrast, Bcl-2expression was 90% lower in shGLOI-transfected SW620 cells (*p* < 0.05), 60% lower in HCT-15 (*p* < 0.001), 50% lower in SW480 (*p* < 0.05), and 40% lower in DLD-1 (*p* < 0.05) than in the shNC-transfected cells (Figure 8A–E), and c-Myc expression was 50% lower in shGLOI-transfected SW480 cells (*p* < 0.05), 60% lower in SW620 (*p* < 0.01), 60% lower in HCT-15 (*p* < 0.001), and 50% lower in DLD-1 (*p* < 0.05) than in the shNC-transfected cells (Figure 8A–E).

### 2.5. Inhibition of GLOI Expressionreduced Tumor Growth in BALB/Cnude Mice

**Figure 9:**
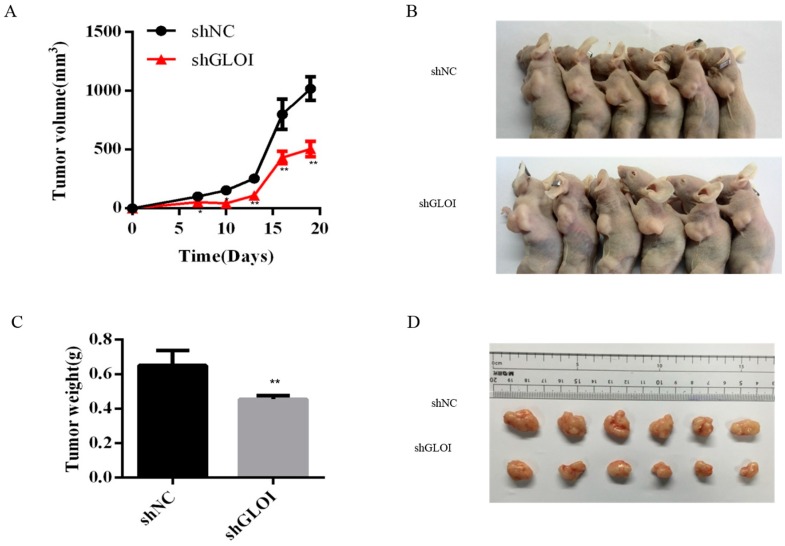
GLOI knockdown reduces SW620 xenograft tumor growth in BALB/c nude mice (6 mice/group). Mice were injected subcutaneously with SW620 colorectal cancer cells transfected with shGLOI or shNC and monitored for SW620 tumor growth beginning on day 7 after injection. Tumors were measured every 3 days from that day. (**A**) Changes in tumor volume over time were plotted. (**B**) The tumor-bearing mice were killed on day 19 and their tumors dissected. (**C**) The weight of each dissected tumor was measured at the time of the animal’s death. All data are representative of three independent experiments (*n* = 6). (**D**) The dissected tumors are shown. ** *p* < 0.01.

**Figure 10:**
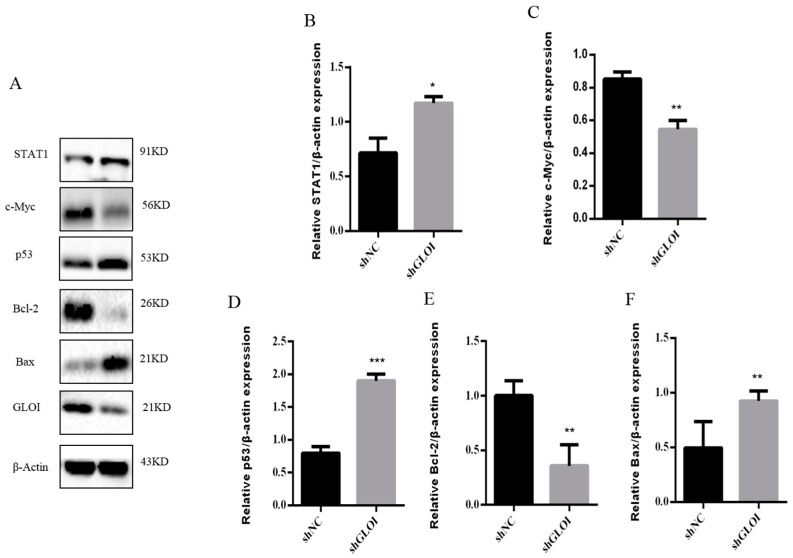
GLOI knockdown alters expression of STAT1 or p53 signal pathway proteins in SW620 colorectal tumors. Tumors were generated by injecting BALB/c nude mice with SW620 cells transfected with shGLOI or shNC (empty vector). (**A**) The expression of STAT1 or p53 signal pathway proteins and GLOI in the tumors were analyzed by western blot. (**B**–**F**) The expression of each protein was quantified and graphed for comparison. * *p* < 0.05, ** *p* < 0.01, *** *p* < 0.001. All data are representative of three independent experiments (*n* = 3).

**Figure 11:**
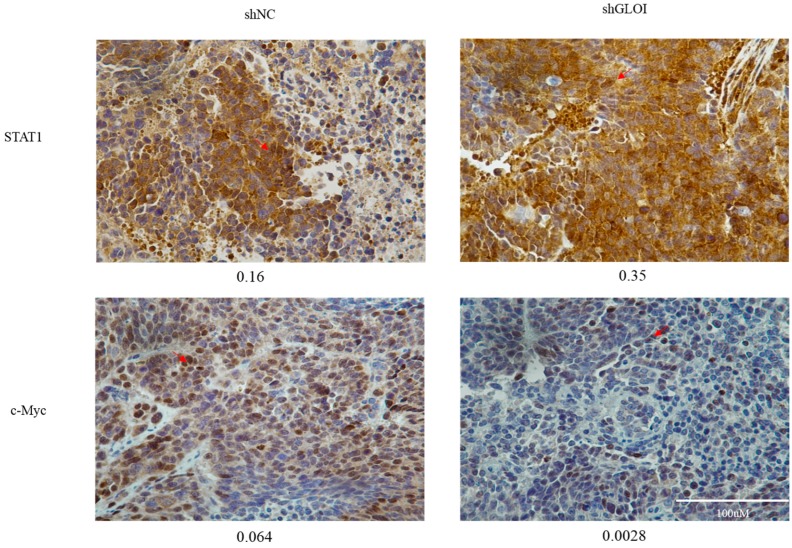
GLOI knockdown alters STAT1 and c-Myc expression in SW620 colorectal xenograft tumors. STAT1 and c-Myc expression was determined by immunohistochemical staining of SW620 tumors generated by injecting BALB/c nude mice with SW620 cells transfected with shGLOI or shNC (empty vector). Representative images are shown (original magnification, ×400). The arrow is pointing at positive cells. The integrated optical density (IOD) of STAT1 and c-Myc positive cells was determined in the CRC tumor tissue and indicated under each image. The arrow pointed at STAT1 or c-Myc expression positive cells in the CRC tumor tissue.

Inoculation with GLOI-depleted SW620 cells induced a significantly lower tumor burden in BALB/c nude mice than inoculation with shNC-transfected cells (Figure 9). The weights of tumors produced by shGLOI-transfected cells dissected on day 19 after inoculation were on average 60% lower than the weights of the tumors from the shNC group (*p* < 0.01) (Figure 9). Expression of STAT1 in GLOI-depleted tumors was 2-fold higher (*p* < 0.05) than in tumors from the shNC group (Figure 10A,B). Similarly, levels of p53 and Bax expression were 2.3-fold and 2.3-fold higher, respectively, in tumors generated by GLOI-depleted cells than in tumors generated by shNC-transfected cells, while c-Myc and Bcl-2 expression levels were 40% and 60% lower, respectively (*p* < 0.01 to 0.001) (Figure 10A,C–F). Immunohistochemical staining also showed higher IOD of expression of STAT1 and lower IOD of expression of c-Myc in tumors produced from GLOI-depleted cells than in tumors produced from shNC-transfected cells (Figure 11).

## 3. Discussion

Our findings show that GLOI was overexpressed in the CRC cell lines tested. GLOI depletion inhibited the proliferation, colony formation, migration, and invasion of CRC cells and induced greater apoptosis of these cells compared with controls. GLOI depletion upregulated the levels of STAT1, p53, and Bax and downregulated c-Myc and Bcl-2 in the CRC cells in vitro. Moreover, the growth of CRC tumors induced by GLOI-depleted SW620 cells in BALB/c nude mice was significantly attenuated. These tumors had higher expression of STAT1, p53, and Bax and lower expression of c-Myc and Bcl-2 than tumors produced from control SW620 cells.

GLOI, which belongs to the glyoxalase system, is an enzyme that catalyzes methylglyoxal metabolism, an intermediate product that mainly produced by glycolysis. Our study demonstrated that GLOI was over-expressed in the CRC cells. GLOI depletion inhibited the proliferation, colony formation, migration, and invasion of CRC cells and induced greater apoptosis of these cells compared with controls. Sakellariou et al. demonstrated that GLOI was overexpressed in colorectal tumor tissue and CRC cell lines. It seems that CRCs overexpressing GLOI gain an advantage by escaping methylglyoxal-induced apoptosis resulting in tumor aggressiveness and low patient survival rate [24]. Hiroshi et al. showed that GLOI activity was elevated in colon cancer cells [18]. In other studies, overexpression of GLOI promoted gastric tumor cell growth and proliferation [19], and inhibition of GLOI expression suppressed tumor cell proliferation [25,26]. A previous study from our laboratory showed that downregulation of GLOI inhibited the migration and invasion of breast cancer cells [9]. In another published report, downexpression of GLOI in gastric cancer cells reduced their migration and invasion activities [27]. Moreover, Hiroya et al. demonstrated that knockdown of GLOI induced apoptosis in cancer cells [28]. Antognelli et al. reported that GLOI inhibition induced apoptosis in irradiated MCF-7 cells [29]. All of these findings are consistent with our results.

In contrast, a recent study found tumors high-stage CRCs with low GLOI expression expression and enzymatic activity when compared with low stage [30]. However, GLOI activity was not compared to the normal tissue in this study. There is a good agreement that GLOI is overexpressed in most malignant tumor as demonstrated by us and many studies [9,10,15,19,25,27]. Additionally, one study suggested that GLOI was overexpressed in colon tumor tissue compared to normal tissue [24]. Further study indicates that GLOI is a novel metabolic oncogene of the 6p21 amplicon, which promotes tumor growth [19]. Different cell lines used in the studies may also be attributed to the ambivalent finding. The mechanisms underlying this controversial finding warrant further investigation.

Investigation of the mechanisms underlying the antitumor effect of GLOI depletion in CRC revealed that GLOI depletion increased STAT1, p53, and Bax expression and decreased c-Myc and Bcl-2 expression in both CRC cells and xenograft tumors in mice. This suggests a novel mechanism by which GLOI depletion restrains tumorigenesis through up-regulation of STAT1. The STAT1 protein, composed of 750 amino acids with a size of 91 kDa, has been classified as a tumor suppressor [31,32,33,34,35,36]. STAT1 is involved in defense against pathogens and inhibition of cell proliferation [37]. Recent studies have reported that a stimulus such as ischemia, heat, DNA damage, or oxysterol is required for STAT1 to promote apoptosis [33]. As a key transcription factor activated during stress-response signaling, STAT1 has been shown to induce apoptosis by upregulating p53 and enhancing the transcriptional activity of p53 on several p53-responsive pro-apoptotic genes such as Bax [33,35,36]. Moreover, recent studies showed that STAT1 negatively regulates the Bcl-xL promoter and is involved in the regulation of all-trans-retinoic acid (ATRA)-induced G0/G1 arrest through downregulation of c-Myc [33,38]. Glyoxalase I inhibition induces apoptosis in irradiated MCF-7 cells via a novel mechanism involving heat shock protein 27 (Hsp27), p53 and nuclear factor-κ B (NF-κB).

**Figure 12:**
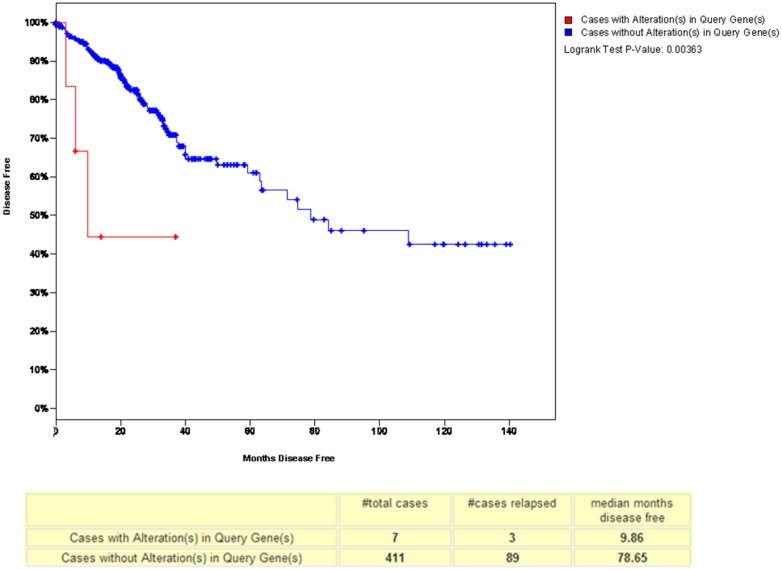
GLOI overexpression shortened disease-free survival in a reference patient cohort from www.cbioportal.org. The red line represents GLOI overexpression levels, the blue line represents GLOI low expression levels.

To analyze the relationship between GLOI and disease-free survival in CRC, we used a tool in www.cbioportal.org [39]. This analysis showed that the patients with a low-GLOI-expressing tumor had longer disease-free survival than the patients whose tumor expressed a high level of GLOI (*p* = 0.00366) (Figure 12). This finding was supported by another report demonstrating that the 5-year survival rate of groups whose tumor expressed a lower level of GLOI was significantly greater than that of the groups whose tumor expressed a higher level of GLOI in gastric cancer [14]. Evidence suggests that GLOI overexpression predicted shortened survival in the entire cohort and in early-stage cases in CRC. Thus, GLOI emerged as an independent prognosticator of adverse significance in the CRC patient cohort [24].

In conclusion, GLOI is overexpressed in CRC cell lines, and GLOI depletion inhibits the tumorigenic properties of these cells both in vitro and in vivo. These tumorigenic effects of GLOI are mediated at least in part by the STAT1 or p53 signaling pathway. Taken together, these findings indicate that GLOI and its related pathways could be a therapeutic target in CRC.

## 4. Materials and Methods

### 4.1. Cell Lines and Cell Culture

CRC cell lines SW480 (Dukes type B), SW620 (Dukes type C), DLD-1 (Dukes type C), and HCT-15 (Dukes type C), representing different pathological stages of CRC, were purchased from the Institute of Biochemistry and Cell Biology, Chinese Academy of Sciences (Shanghai, China). Normal colon FHC cells were obtained from the same source. All cells were cultured in RPMI-1640 medium (Gibco, Carlsbad, CA, USA) supplemented with 10% fetal bovine serum (FBS; Bioind, Beit-Haemek, Israel) and 100 U/mL penicillin-streptomycin (P1400; Solarbio, Beijing, China) in a humidified 37 °C atmosphere supplemented with 5% CO_2_.

### 4.2. Cell Transfection

The short hairpin RNA sequences targeting GLOI and empty vector were synthesized by GenePharma (Shanghai, China). The sequences of short hairpin GLOI (shGLOI) were 5′-GGATTCGGTCATATTGGAATT-3′; 5′-GGGAGTCAAATTTGTGAAGAA-3′; 5′-GGCATTTATTCAAGATCCTGA-3′; and 5′-GAAGAACTGGGAGTCAAATTT-3′. SW480, SW620, DLD-1, and HCT-15 cells were transfected after they had grown to 80%–90% confluence. Transfection with shGLOI or short hairpin empty vector (shNC, negative control) was carried out with Lipofectamine 2000 (Invitrogen, Shanghai, China) according to the manufacturer’s instructions (DNA to Lipofectamine 2000 ratio, 4:5). Six hours after transfection, the culture medium was replaced with fresh RPMI-1640 medium containing 10% FBS. Twenty-four hours after transfection, cells were examined under a fluorescence microscope to determine transfection efficiency. After transfection, stably GLOI-over-expressing cells (transfection efficiency ≥ 50%) were established by incubating cells in complete RPMI-1640 medium containing 1000 μg/mL G418 (11811031; Sigma, St. Louis, MO, USA) for 15 days. The clones were verified by western blot, real-time quantitative reverse-transcriptase polymerase chain reaction (qRT-PCR), and quantification of GLOI enzymatic activity. The successful clones were pooled and used for further investigations.

### 4.3. Quantification of GLOI mRNA

Total RNA was extracted from transfected CRC cells by using TRIzol (3101-100; Invitrogen) according to the manufacturer’s instructions. Total RNA (500 ng) was reverse-transcribed to cDNA with random primer (RRO37A; Takara Bio, Shiga, Japan). Gene expression was measured by reverse transcription PCR (RT-PCR) using an Applied Biosystems 7500 Fast Sequence Detection System and SYBR Green PCR Kit (20454; Qiagen, Shanghai, China) under the following conditions: denaturation at 95 °C for 5 min, followed by 40 cycles of denaturation at 95 °C for 10 s and annealing and extension at 60 °C for 30 s. The GLOI mRNA expression was normalized to that of glyceraldehyde-3-phosphate dehydrogenase (GAPDH) Primers (Sangon Biotech, Shanghai, China) for GLOI were 5′-AGCAGACCATGCTACGAGTG-3′ (forward) and 5′-TAGCTTTTCTGGAGAGCGCC-3′ (reverse). Primers for the internal control GAPDH were5′-AAGGTGAAGGTCGGAGTCAAC-3′ (forward) and 5′-GGGGTCATTGATGGCAACAATA-3′ (reverse).

### 4.4. Western Blotting Analysis

Cells were transfected with shGLOI or shNC, harvested, and subjected to lysis by a protease inhibitor cocktail and then to centrifugation at 14,000× *g* for 15 min at 4 °C. The supernatant fraction was collected and the protein concentration was measured by using a bicinchoninic acid protein assay kit (Beyotime, Hangzhou, China). An aliquot (40 μg) of denatured protein from each sample was treated with sodium dodecyl sulfate and applied to a 12% polyacrylamide gel for electrophoretic separation, then transferred onto a nitrocellulose membrane. After blocking with 5% nonfat milk for 2 h at room temperature, membranes were incubated at 4 °C overnight with the following primary antibodies (1:1000 dilution): anti-GLOI antibody, anti-Bcl-2 antibody, anti-Bax antibody, anti-c-Myc antibody, anti-signal transducer and activator of transcription 1 (anti-STAT1) antibody (all from Abcam, Cambridge, UK), and anti-β-actin antibody (Beyotime, Hangzhou, China). Membranes were washed with Tris-buffered saline/0.05% Tween-20 solution (TBST) and incubated with horseradish peroxidase–conjugated secondary antibody (1:2000 dilution) for 1 h at room temperature. After washing with TBST for 1 h, the blots were incubated with chemiluminescent substrate, Western Bright Quantum (AdvanstaK-12045-D10, Shanghai, China) as recommended by the manufacturer. The blots were imaged using a charge coupled device (CCD) imager and signals were quantified by densitometry. β-Actin was used as an internal control.

### 4.5. Quantification of GLOI Enzymatic Activity

Cells transfected with shGLOI or shNC were harvested by trypsinization and collected by centrifugation at 650× *g* for 5 min. After two washings with phosphate-buffered saline solution (PBS), treated cells were subjected to lysis buffer [radio-Immunoprecipitation assay (RIPA) lysis buffer to phenylmethanesulfonyl fluoride (PMSF) ratio, 100:1] in the presence of a protease inhibitor cocktail and then to centrifugation at 12,000× *g* for 15 min at 4 °C. The resulting cell extracts were assayed for GLOI activity with the Quantichrom Glyoxalase I Assay Kit (Shanghai Universal Biotech Co., Shanghai, China).

### 4.6. Cell Proliferation Assay

Cell proliferation was assessed by using the Cell Counting Kit-8 (CCK-8) Kit (CK04; Dojindo, Kumamoto, Japan) [40]. SW480, SW620, DLD-1, and HCT-15 cells stably transfected with shGLOI or shNC were seeded in 96-well plates at a density of 3 × 10^3^ cells/well, 2.5 × 10^3^ cells/well, 3 × 10^3^ cells/well, or 2 × 10^3^ cells/well, respectively, and incubated for various periods of time (0 to 5 days). Following incubation, Cell Counting Kit-8 (CCK-8) assay reagent (10 μL) was added to each well and the plates incubated at 37 °C for 1 h. Absorbance was measured at 450 nm using an Multiskan Spectrum reader (Thermo Scientific, Waltham, MA, USA).

### 4.7. Colony Formation Assay

The plate colony formation assay was used to detect cell growth [40]. All cells were seeded at 3 × 10^3^ cells/well in 6-well plates. After 15 days, colonies could be observed directly with the unaided eye. The colonies were fixed with 4% paraformaldehyde for 15 min and stained with crystal violet for 15 min at ambient temperature. After washing twice with PBS, the colonies were viewed and counted under a microscope at ×40 magnification. Only clearly visible colonies (diameter > 50 µm) were counted.

### 4.8. Cell Migration Assay

CRC cells were seeded in 6-well plates. The cells were then collected and washed twice with PBS and resuspended with serum-free 1640 medium and seeded (SW480, 8 × 10^4^ cells; SW620, 1.2 × 10^5^ cells; DLD-1, 8 × 10^4^ cells; HCT-15, 8 × 10^4^ cells) into the upper chambers of transwell culture plates, each well lined with an 8-μm pore membrane insert (Corning, Shanghai, China). RPMI-1640 medium supplemented with 20% FBS was placed in the lower chambers as a chemoattractant. The SW480, SW620, DLD-1, and HCT-15 cells were incubated for 32, 48, 24, and 24 h, respectively. Cells that penetrated through to the lower surface of the membranes were fixed with 4% paraformaldehyde for 20 min and stained with crystal violet (C0121; Beyotime, Hangzhou, China) for 20 min at ambient temperature. They then were counted under a microscope (Nikon, Tokyo, Japan) at ×200 magnification in five randomly chosen fields.

### 4.9. Cell Invasion Assay

The cell invasion assay was similar to the migration assay except that the transwell chambers were coated with matrigel solution (45 μL per chamber; matrigel: serum-free medium ratio, 1:10), and all steps were performed on ice. SW480 cells (1 × 10^5^ cells/well), SW620 cells (1.6 × 10^5^ cells/well), DLD-1 cells (1 × 10^5^ cells/well), or HCT-15 cells (1 × 10^5^ cells/well) were seeded into the upper chambers of the transwell plates and RPMI-1640 medium with 20% FBS (600 μL) was added into the lower chambers as a chemoattractant. After 48 h, the cells that had penetrated the matrigel and moved to the lower surface of the membrane were fixed with 4% paraformaldehyde and stained with crystal violet. Cells adhering to the upper surface of the membrane were removed with a cotton swab. The cells attached to the lower surface were counted under a microscope (Nikon, Tokyo, Japan) at ×200 magnification in five randomly chosen fields.

### 4.10. Quantification of Cellular Apoptosis

Cell apoptosis was detected using the Annexin V-APC Apoptosis Detection kit (KeyGEN Biotech, Nanjing, China) as described in our previous studies and others [41,42,43,44]. Allophycocyanin (APC) is an accessory photosynthetic pigment found in blue-green algae. APC has 6 phycocyanobilin chromophores per molecule, which are similar in structure to phycoerythrobilin, the chromophore in phycoerythrin or PE. In brief, the cells were seeded in 6-well plates (6 × 10^5^ cells/well). After incubation for 24 h, cells were collected and washed twice with PBS. The cell suspensions were stained with annexin V and Propidium iodide (PI) from the kit for 30 min at 4 °C in the dark. Cell apoptosis was determined as either annexin positive or both annexin and PI positive by flow cytometry and the percentage of apoptotic cells was calculated.

### 4.11. Tumor Xenografts in Mice

Twelve male athymic BALB/c nude mice (4 weeks old) were obtained from the Shanghai Medical Experimental Animal Care Commission (Shanghai, China). All animal procedures and experimental protocols were approved by the Laboratory Animal Ethics Committee of Wenzhou Medical University (wydw2015-0120, 6 March 2015).

To establish xenograft tumors, SW620 cells stably transfected with shGLOI (4 × 10^6^ in 200 uL of medium) were injected subcutaneously into the dorsal flank of each mouse. Other mice were injected with SW620 cells stably transfected with shNC as negative controls. Each mouse’s tumor was measured every 3 days starting from day 7 after the injection, using a Vernier caliper along two perpendicular axes. The volume of the tumor was calculated using the formula: volume = (length × width^2^)/2. Nineteen days after subcutaneous injection, the mice were killed and the tumors were dissected for analyses.

Total RNA was extracted from a section of each dissected xenograft tumor by the same method used for CRC cell lines in vitro. The dissected tumors were also subjected to western blot analysis for GLOI and other cellular proteins as already described for CRC cells in vitro.

### 4.12. Immunohistochemical Analysis

Tumor tissues were subjected to immunohistochemical analysis for STAT1 and c-Myc with a commercial kit (Boster, Wuhan, China) used according to manufacturer’s instructions. Briefly, every tissue section was formalin-fixed, paraffinembedded, deparaffinized, then rehydrated and rinsed with PBS. For the high-pressure antigen, retrieval was carried out in citrate buffer, which was then removed by rinsing with PBS. The concentration of citrate buffer salt was 0.01 M (pH 6.0). Trisodium citrate (C_6_H_5_Na_3_O_7_·2H_2_O) 3 g and citric acid (C_6_H_8_O_7_·H_2_O) 0.4 g were dissolved in 1 L of distilled water. The sections were incubated with endogenous peroxidase blockers for 15 min in the dark and then with 5% bovine serum albumin for 30 min at 37 °C. The sections were then sequentially incubated with specific primary antibody (anti-c-Myc, anti-STAT1), biotinylated goat anti-rabbit IgG, and avidin-biotin peroxidase complex, and rinsed with PBS. The slides were stained with 3,3-diaminobenzidine, counterstained with hematoxylin, and photographed. The antigen signals on each slide were counted under a microscope (Nikon, Tokyo, Japan) at ×400 magnification. The integrated optical density (IOD) of STAT1- or c-Myc-positive cells in the CRC tumor tissue after immunohistochemical staining was determined by image Pro Plus (Media Cybernetics, Bethesda, MD, USA).

### 4.13. Statistical Analyses

Data are summarized as the means ± standard deviation for all samples, calculated with SPSS 17.0 software (IBM, Armonk, NY, USA). Differences between groups were analyzed by using analysis of variance and the two-tailed Student *t*-test. *p*-Values of <0.05 were considered statistically significant. All experiments were performed independently in triplicate.

## Abbreviations

CCK-8	Cell Counting Kit-8
CRC	Colorectal cancer
FBS	Fetal bovine serum
GLOI	GlyoxalaseI
PBS	Phosphate-buffered saline solution
qRT-PCR	Quantitative real-time PCR
STAT1	Signal transducer and activator of transcription 1
